# The effect of small incision lenticule extraction on contrast sensitivity

**DOI:** 10.3389/fnins.2023.1132681

**Published:** 2023-04-12

**Authors:** Pinqing Yue, Zeng Wang, Di Wu, Hua Zhang, Pan Zhang

**Affiliations:** ^1^Department of Psychology, Hebei Normal University, Shijiazhuang, China; ^2^Department of Psychology, Hebei Medical University, Shijiazhuang, China; ^3^Department of Medical Psychology, Air Force Medical University, Xi’an, China; ^4^Department of Ophthalmology, Shijiazhuang People’s Hospital, Shijiazhuang, China

**Keywords:** small incision lenticule extraction, contrast sensitivity, spatial frequency, external noise, perceptual template

## Abstract

The improvements due to small incision lenticule extraction (SMILE) in vision, e.g., in spherical equivalent (SE) and visual acuity (VA), has been widely recognized. However, the contrast sensitivity (CS) change after SMILE was not certain. Here, we investigated the effect of SMILE on CS before, 1 day after and 7 days after surgery and then clarified the corresponding mechanism by using a perceptual template model (PTM). In addition, the relationship among SE, VA, and CS was discussed. The quick contrast sensitivity function (qCSF) was applied to measure CS with high precision and accuracy. We found that (1) CS was significantly improved 1 day after SMILE and was also increased 7 days after the surgery, (2) CS improvements were dependent on spatial frequency and external noise, (3) the increase in CS was due to the decreased internal additive noise and an enhanced perceptual template, and (4) Greater SE improvements predicted better VA improvements 1 day after SMILE, and a positive correlation between SE improvements and AULCSF improvements 7 days after SMILE was observed. These findings help us better understand the effect of SMILE and provide effective indicators for future visual research.

## Introduction

Myopia has become a global issue. To correct myopia, patients are increasingly inclined to have corrective surgery, especially small incision lenticule extraction (SMILE), which has been proven to be a safe, effective and stable method to improve visual performance (Ganesh and Gupta, 2014; Vestergaard et al., 2014; Chansue et al., 2015; Ganesh et al., 2018); for example, nearsighted people noted that it became easier to drive cars after treatment (Klokova et al., 2019). In addition, compared with other refractive surgery methods, SMILE has unique advantages, including minimal invasiveness and fewer sequelae (Han et al., 2020; Huang and Melki, 2021). Recent studies have focused on the selection of visual indicators to evaluate the effects of SMILE.

Two indicators, diopter (D) and visual acuity (VA), are the characteristics most commonly used in studies that assess visual function after SMILE: D is a measure of the refractive error, and myopia is defined as ≤−0.50 D (Sankaridurg et al., 2021); VA is a measure of the ability to resolve fine detail (Zimmerman et al., 2011). Notably, few studies have introduced contrast sensitivity (CS) as a variable of visual performance together with VA. CS refers to the ability to detect a target at various spatial frequencies (SFs); however, VA is estimated under high contrast (at least 85%) and high SF (Owsley, 2003). CS is more sensitive in disease diagnosis (e.g., cataract and glaucoma) than VA (Hou et al., 2010; Shakarchi et al., 2019). Hence, CS should be taken into account when evaluating the effects of SMILE on myopia.

Until now, how CS changes after SMILE remained uncertain. For example, using the CSV-1000 test, Gertnere et al. (2013) found that CS improved after surgery; however, in another study (Liu et al., 2016), postoperative and preoperative CS were comparable. However, there may have been some issues with the contrast sensitivity function (CSF) measurement procedure they used. For example, the SF of gratings in CSV-1000 was only four, and the data obtained were inadequate to draw the CSF. Fortunately, the quick CSF (qCSF) method proposed by Lesmes et al. (2010) can solve the problem well. The qCSF concurrently estimates thresholds across the full SF range and then obtains a complete CSF in 50 trials with a combination of good accuracy and precision (Hou et al., 2016). In the current study, we used the qCSF to accurately and precisely evaluate the effect of SMILE on visual function.

Second, prior studies hardly accounted for the influence of external noise. The complexity of the real-world environment can affect visual performance. For example, bad weather conditions (e.g., snowing) limit a driver’s vision and pose potential driving risks (Horswill and Plooy, 2008; Zang et al., 2019). The external noise paradigm is often combined with the perceptual template model (PTM) to explain the underlying mechanisms of visual perception (McAnany and Alexander, 2010; Allard et al., 2013). The PTM can decompose the limitations of visual perception into three independent mechanisms (Lu and Dosher, 2008): (1) internal additive noise, which can amplify or weaken both signal and noise from input stimuli, (2) internal multiplicative noise, which describes the consequences of contrast gain control systems, and (3) perceptual template, i.e., the ability to exclude external noise. Explaining the effect of SMILE on visual function by the PTM is very interesting.

In brief, the objectives of this study included (1) determining how SMILE affects D, VA, and CS, (2) examining whether or not CS increase depends on SF, external noise, and recovery time, (3) investigating the possible relation among improvements in vision indicators, and (4) revealing the relevant mechanisms using the PTM.

## Methods

### Participants

To determine the required sample size for this study, we used G*Power 3.1.9.7 (Faul et al., 2007). Based on a moderate effect size (*f*) = 0.25, *α* = 0.05, and the power of the tests (1 − *β*) = 0.95, the required sample size for a repeated-measures analysis of variance (ANOVA) was six. We recruited fourteen participants (aged 18 years or older) from Shijiazhuang People’s Hospital. Before the surgery, they all voluntarily signed informed consent for data collection. Patients were included when they maintained stable refraction for at least 1 year, used soft contact lenses but discontinued use for 1 week or used rigid gas permeable lenses but discontinued use for 3 weeks prior to the procedure; their minimum corneal thickness was 480 μm and residual stromal bed was at least 250 μm. Patients were not included if they had other serious eye diseases except myopia and astigmatism. This study was authorized by the Ethics Committee of Hebei Normal University and the Ethics Committee of Shijiazhuang People’s Hospital. We adhered to the tenets of the Declaration of Helsinki.

### Apparatus

All stimuli were produced by MATLAB + PsychToolbox and presented on a luminance-calibrated Apple (CRT) monitor with a resolution of 1,280 × 1,024, a refresh rate of 85 Hz and 36.3 cd/m^2^ background brightness. The viewing distance was 176 cm. Only one eye of each participant was tested.

### Stimuli

The experiment consisted of target stimuli and masks: target stimuli were vertical gratings at 10 SFs (0.5, 0.67, 1, 1.33, 2, 2.67, 4, 5.33, 8, and 16 cpd). All gratings were displayed for three cycles, and they were covered with truncated Gaussian envelopes to blur their edges. Masks were noise images that were obtained from Gaussian distributed pixel contrasts and were composed of the same number of noise elements (15 × 15). In each trial, the sizes of the signal and noise images were identical.

### Procedure

Spherical equivalent (SE) refraction, which was measured by a TOPCON RM-8900 autorefractor, was the indicator of D, and it was equal to spherical power (DS) plus half the cylinder power (DC). VA was measured by using the E-chart at a distance of 5 m and was recorded as the logMAR value for statistical analyses. The smaller the logMAR value was, the better the VA outcome was.

**Figure 1:**
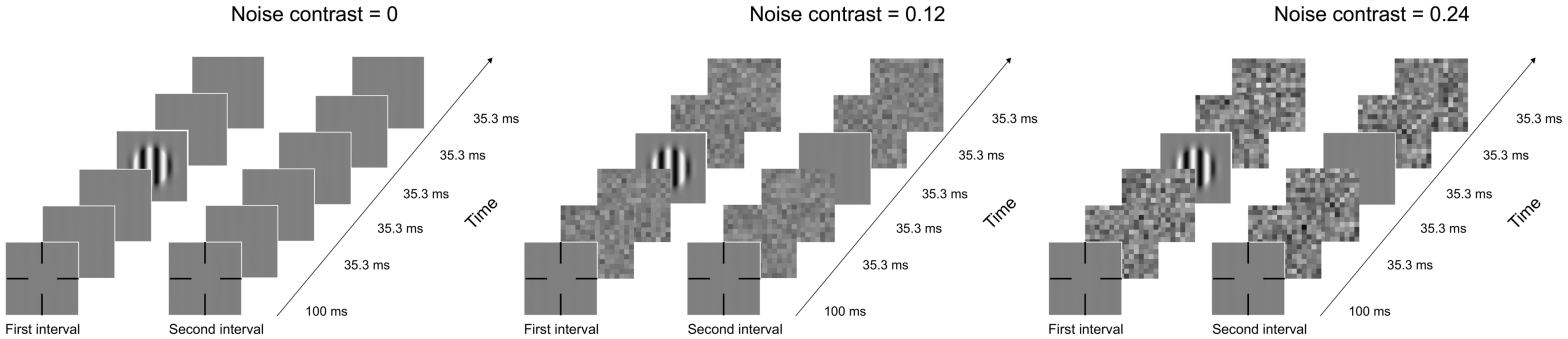
Illustration of a typical trial under zero- (left), low- (medium), and high- (right) noise conditions. Each condition has two intervals, and every interval has five images, including one blank or grating image and four noise images. When external noise was absent, noise images were replaced by blanks.

The qCSF method was used to measure the CSF (Figure 1). Each trial included two intervals and the presentation of a blank screen for 500 ms between them. The grating was shown in the first or second interval. Participants were required to identify and react to the grating by using a gamepad. Each interval consisted of five images presented for 35.3 ms. In the noise condition, one blank or grating image was sandwiched by four noise images. In the noise-free condition, the four noise images were replaced by blanks. A whole CSF measurement contained 10 SFs and 3 external noise levels. Three external noise levels were randomly presented across trials, and each noise level consisted of 50 trials.

### Design

The whole experiment was divided into 3 stages: pretest, posttest 1 (the first day after surgery), and posttest 2 (the seventh day after surgery). Each test included measurements of VA, CS, and SE. VA and SE tests were performed in an illuminated room with the lights on. After a 5 min period of dark adaptation, CS was assessed by the qCSF method within 15 min.

### Statistical analyses

Repeated-measures ANOVA was used to analyze the influence of time point, external noise, and SF on CS and its improvements; repeated-measures ANOVA was used to analyze the influence of time point and external noise on the area under the log CSF (AULCSF) and its improvements; least significant difference (LSD) test was used for multiple comparisons; and Pearson correlation analysis was used to analyze the correlation among SE, VA, and CS.

The PTM was applied to effectively clarify the potential mechanisms of CS improvements. First, *d*′ represents the perceptual sensitivity of the patients and is expressed by Eq. (1):


(1) $$d^{\prime }=\frac{{\left(\beta c\right)}^{\gamma }}{\sqrt{{\left(N_{\mathrm{ext}}\right)}^{2\gamma }+N_{\mathrm{mul}}^{2}\left[{\left(\beta c\right)}^{2\gamma }+{\left(N_{\mathrm{ext}}\right)}^{2\gamma }\right]+N_{\mathrm{add}}^{2}}}$$

where *c* is the signal contrast; *β* signifies the perceptual template gain; and *γ* implies the nonlinearity of the system. *N*_add_ is the equivalent internal additive noise, *N*_mul_ is the equivalent internal multiplicative noise, and *N*_ext_ is the contrast of external noise. When *d*′ is a given value, *c_τ_* can be calculated from Eq. (2):


(2) $$\begin{array}{l} c_{\tau }=\frac{1}{2\gamma }\log\left[\left(1+N_{\mathrm{mul}}^{2}\right){\left(N_{\mathrm{ext}}\right)}^{2\gamma }+N_{\mathrm{add}}^{2}\right]\\ \;\;\;\;\;\;\;\;\;\;-\frac{1}{2\gamma }\log\left(\frac{1}{d\prime^{2}}-N_{\mathrm{mul}}^{2}\right)-\log\left(\beta \right) \end{array}$$

Then, for simulating CS changes due to SMILE, *A_a_*, *A_m_* and *A_f_* were utilized to adjust *N*_add_, *N*_mul_ and *N*_ext_, respectively. Therefore, $c_{\tau }$ is expressed as Eq. (3):


(3) $$\begin{array}{l} c_{\tau }=\frac{1}{2\gamma }\log\left[\left(1+A_{m}^{2}N_{\mathrm{mul}}^{2}\right){\left(A_{f}N_{\mathrm{ext}}\right)}^{2\gamma }+A_{a}^{2}N_{\mathrm{add}}^{2}\right]\\ \;\;\;\;\;\;\;\;\;-\frac{1}{2\gamma }\log\left(\frac{1}{d\prime^{2}}-A_{m}^{2}N_{\mathrm{mul}}^{2}\right)-\log\left(\beta \right) \end{array}$$

The multiplicative noise was set as a constant when the slopes of psychometric functions were not altered from pretest to posttest 1 and posttest 2 (Xu et al., 2006; Lu and Dosher, 2008). In addition, *N*_add_ and *β* were dependent on SFs, while *N*_mul_ and *γ* were independent of SFs. In brief, the effects of SMILE on visual perception can be explained by two possible mechanisms: lower internal additive noise and enhanced perceptual template (i.e., elimination of external noise).

## Results

### Spherical equivalent

A repeated-measures ANOVA was used to test SE. The main effect of time point (pretest, posttest 1, and posttest 2) was significant [*F*(2, 26) = 93.526, *p* < 0.001]. The LSD analysis found that SE at posttest 1 and posttest 2 was significantly better than that at pretest (all *p* < 0.001), reflecting the improvements in SE due to SMILE. In addition, due to the ceiling effect, there was no significant difference in SE between posttest 1 and posttest 2 (*p* = 0.787).

### Visual acuity

The result of a repeated-measures ANOVA revealed that SMILE could improve VA effectively. Time point had a significant main effect on VA [*F*(2, 26) = 81.875, *p* < 0.001]. Specifically, the LSD test showed that VA at posttest 1 was significantly better than that at pretest (*p* < 0.001). VA at posttest 2 was also better than that at pretest (*p* < 0.001). Moreover, VA was comparable between posttest 1 and posttest 2, indicating that VA tended to be stable (*p* = 0.051).

### Contrast sensitivity

**Figure 2:**
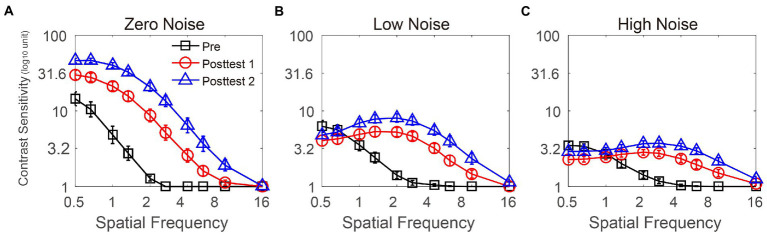
CS functions at three time points (pretest vs. posttest 1 vs. posttest 2) under zero- **(A)**, low- **(B)**, and high- **(C)** noise levels. Black lines with squares, red lines with circles, and blue lines with triangles represent contrast sensitivity at pretest, posttest 1 and posttest 2, respectively. All data were averaged across subjects. Error bars denote standard errors (SEs).

The curves in Figures 2A–C represent CS at three external noises at pretest, posttest 1 and posttest 2; notably, the curves suggested that SMILE enabled CS recovery in all noise conditions. We applied repeated-measures ANOVA to analyze CS with external noise (zero, low, and high), time point (pretest, posttest 1, and posttest 2) and SF (from 0.5 to 16 cpd) as three within-subject factors. The main effects of external noise, time point and SF were significant [*F*(2, 26) = 161.807, *p*< 0.001; *F*(2, 26) = 89.641, *p*< 0.001; *F*(9, 117) = 261.364, *p*< 0.001, respectively]. The two-way interactions between external noise and time point, between time point and SF, and between external noise and SF were all significant [*F*(4, 52) = 33.03, *p* < 0.001; *F*(18, 234) = 33.497, *p* < 0.001; *F*(18, 234) = 131.13, *p* < 0.001, respectively]. The three-way interaction among the above variables was significant [*F*(36, 468) = 12.571, *p* < 0.001]. The LSD test was used to analyze the influence of time points on CS under each SF when noise conditions changed (see Supplementary Table S1). In the zero-noise condition, from 0.5 to 5.33 cpd, CS at posttest 2 was the best, followed by CS at posttest 1 and CS at pretest was the worst (all *p* < 0.013). At 8 cpd, CS at posttest 2 was significantly greater than that at pretest and posttest 1 (*p* = 0.003; *p* = 0.002, respectively); CS was comparable between posttest 1 and pretest (*p* = 0.131). At 16 cpd, there was no difference in CS among the three time points (all *p* > 0.175), which may be due to the floor effect. These analyses suggested that in the zero-noise condition, CS at 0.5–8 cpd could be raised through SMILE, and CS increased gradually with time. In the low noise condition, at 0.5 cpd, CS at posttest 2 was not significantly different from that at pretest and posttest 1 (all *p* > 0.198); CS at pretest was significantly higher than that at posttest 1 (*p* = 0.039). At 0.67 and 8 cpd, there were no significant differences in CS among the three time points (all *p* > 0.082). At 1 cpd, CS at posttest 2 was better than that at posttest and posttest 1 (*p* = 0.003; *p* = 0.033, respectively); no significant difference in CS was detected between pretest and posttest 1 (*p* = 0.136). From 1.33 to 8 cpd, CS at posttest 2 was the best, followed by CS at posttest 1 and CS at pretest was the worst (all *p* < 0.05). At 16 cpd, there were no significant differences among all time points, which may be due to the floor effect. The results indicated that in low noise conditions, the improvements due to SMILE were effective at 1–8 cpd, and postoperative CS improved continuously due to postoperative recovery. In the high noise condition, at 0.5 and 0.67 cpd, CS at pretest and posttest 2 was significantly better than that at posttest 1 (all *p* < 0.003); CS was not significantly different between pretest and posttest 2 (all *p* > 0.097). At 1 cpd, CS at pretest was comparable to that at posttest 1 and posttest 2 (all *p* > 0.131); CS at posttest 2 was greater than that at posttest 1 (*p* = 0.018). From 1.33 to 8 cpd, CS at posttest 2 was the best, followed by CS at posttest 1 and CS at pretest was the worst (all *p* < 0.013). At 16 cpd, CS at posttest 2 was significantly higher than that at pretest (*p* = 0.026); CS at posttest 1 was comparable to that at pretest and posttest 2 (all *p* > 0.172). These data revealed that at 1.33–16 cpd, CS increased gradually. In summary, SMILE improved CS effectively, but the effects were modulated by the time points, SF, and external noise. Specifically, under the three noise conditions, CS increased immediately at 1.33–5.33 cpd (posttest 1 vs. pretest), further improved with recovery from surgery at 1–8 cpd (posttest 2 vs. posttest 1), and generally improved after SMILE at 1.33–8 cpd (posttest 2 vs. pretest). In noise condition, SMILE temporally reduced CS at 0.5 and/or 0.67 cpd. In addition, SMILE was unable to increase CS at the highest SF (16 cpd), which may be due to the floor effect.

To elucidate how the CS improvements were modulated by SF, we calculated it at posttest 1 (CS at posttest 1 minus that at pretest) and posttest 2 (CS at posttest 2 minus that at pretest). A repeated-measures ANOVA was conducted to assess CS improvements with three within-subject factors (time point, SF, and external noise). The results showed that the main effects of time point, SF, and external noise were significant [*F*(1, 13) = 56.844, *p* < 0.001; *F*(9, 117) = 48.664, *p* < 0.001; *F*(2, 26) = 44.686, *p* < 0.001, respectively]; two-way interactions between time point and SF, between time point and external noise, and between SF and external noise were significant [*F*(9, 117) = 5.565, *p* < 0.001; *F*(2, 26) = 10.962, *p* < 0.001; *F*(18, 234) = 19.653, *p* < 0.001, respectively]; additionally, three-way interaction was significant [*F*(18, 234) = 1.891, *p* = 0.017]. Additionally, The LSD tests revealed that the CS improvements first increased with SF, then after 2 or 2.67 cpd, it decreased with SF. Detailed *p* values of comparison were listed in Supplementary Table S2.

**Figure 3:**
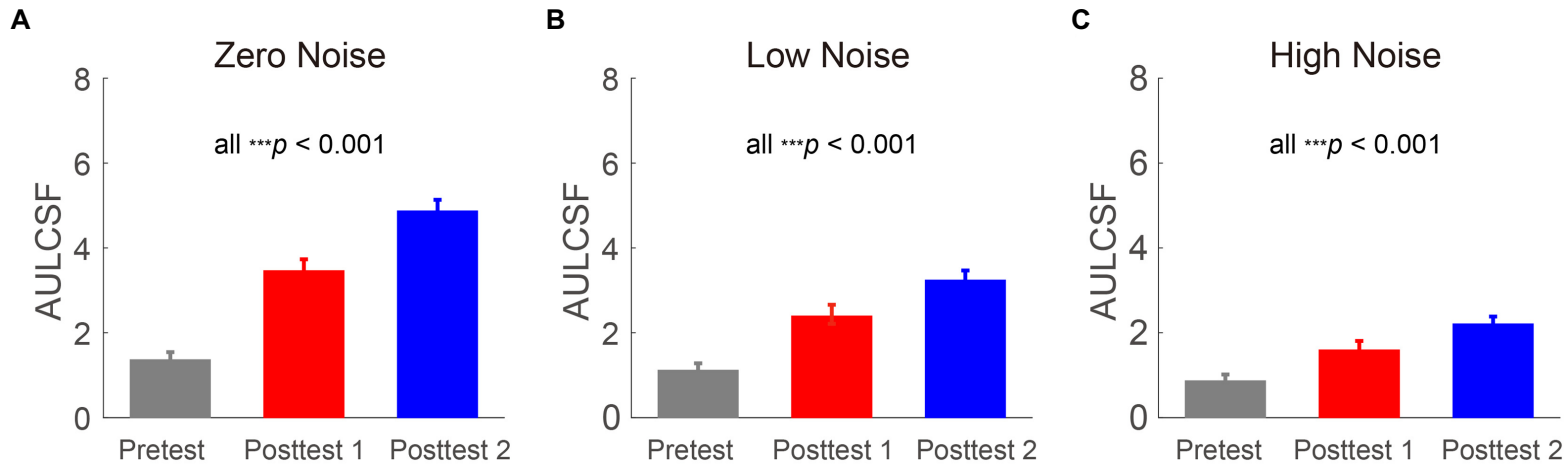
Areas under log CS functions (log_10_ units) at three time points (pretest vs. posttest 1 vs. posttest 2) in zero- **(A)**, low- **(B)**, and high- **(C)** noise conditions. Gray, red and blue bars represent the results at pretest, posttest 1 and posttest 2, respectively. All data were averaged across subjects. Error bars denote standard errors (SEs).

To measure the effect of SMILE on CSF over all SFs, the AULCSF was calculated as shown in Figures 3A–C. A repeated-measures ANOVA was performed to assess AULCSF data with time point (pretest, posttest 1, and posttest 2) and external noise (zero, low, and high) as two within-subject variables. The main effects of time point and external noise and the interaction among them were all significant [*F*(2, 26) = 90.074, *p* < 0.001; *F*(2, 26) = 121.969, *p* < 0.001; *F*(4, 52) = 30.399, *p* = 0.037, respectively]. The LSD tests revealed that among the three external noise conditions, AULCSF at posttest 2 was the best, followed by CS at posttest 1 and CS at pretest was the worst (all *p* < 0.001), indicating that AULCSF increased over time.

To explore whether the AULCSF improvements were dependent on external noise. They were computed at posttest 1 (AULCSF at posttest 1 minus that at pretest) and posttest 2 (AULCSF at posttest 2 minus that at pretest). Repeated-measures ANOVA was performed on the AULCSF improvements with time point (posttest 1 and posttest 2) and external noise (zero, low, and high) as two within-subject variables. The main effects of time point and external noise and the interaction between them were all significant [*F*(1, 13) = 58.454, *p* < 0.001; *F*(2, 26) = 41.402, *p* < 0.001; *F*(2, 26) = 11.271, *p* < 0.001, respectively]. The LSD tests showed that at two time points, AULCSF improvements decreased as noise increased (all *p* < 0.014), indicating the external noise depended AULCSF improvements. Among the three external noise conditions, AULCSF improvements at posttest 2 were significantly larger than those at posttest 1 (all *p* < 0.001), indicating that the recovery of CS required time. These findings indicated that AULCSF improvements were dependent on time point and external noise, with larger improvements observed as postoperative recovery progressed and at lower external noise levels.

**Figure 4:**
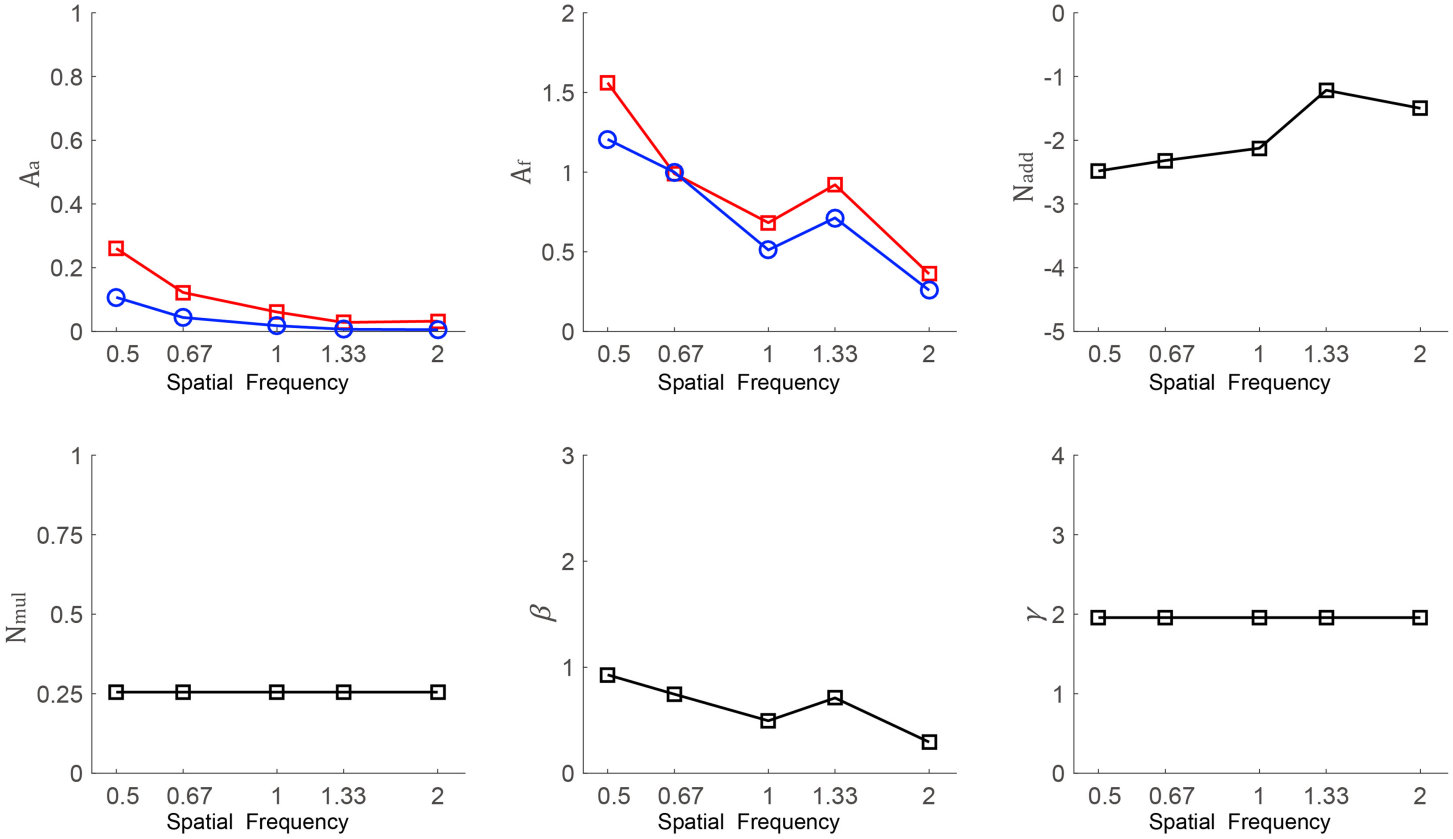
*A_a_*, *A_f_*, *N*_add_, *N*_mul_, *β*, and *γ* as a function of SFs from the best-fitting model. The red line with squares and the blue line with circles indicate data from posttest 1 and posttest 2, respectively.

To recognize the mechanisms underlying the postoperative CS improvements, the average data of all observers was fitted to the PTM (as shown in Figure 4). As mentioned above, we assumed that multiplicative noise was constant when slopes of psychometric functions were independent of the time point. To prove the hypothesis, we conducted a repeated-measures ANOVA on slopes with time point (pretest vs. posttest 1 vs. posttest 2) as a within-subject factor and found that slopes were unchanged with time, which supported the above assumption [*F*(2, 26) = 1.997, *p* = 0.156]. Thus, we removed *A_m_* from Eq. (3). There were four models: the full model, which hypothesized that SMILE decreased internal additive noise (*A_a_* < 1) and enhanced the perceptual template (*A_f_* < 1); reduced model 1 (M_1_), which assumed that SMILE decreased internal additive noise (*A_a_* < 1); reduced model 2 (M_2_), in which SMILE was considered to improve the perceptual template (*A_f_* < 1); and the most reduced model (M_3_), in which none of the parameters were changed by SMILE. The best-fitting model had to satisfy both conditions of a comparable *r^2^* value with the full model and the fewest free parameters covered. From the full model to the most reduced model, in order, the *r^2^* values were 91.8% (*A_a_* and *A_f_* change), 60.6% (*A_a_* change), 37.9% (*A_f_* change), and 33.0% (no change). An F test was used to compare models. The *r^2^* of the full model was significantly better than that of M_1_, M_2_ and M_3_ [*F*(10, 238) = 90.597, *p* < 0.001; *F*(10, 238) = 1.566, *p* < 0.001; *F*(12, 238) = 85.261, *p* < 0.001, respectively]. Thus, the full model was determined to be the best-fitting model. To explore the relationship among SF, *A_a_*, and *A_f_*, we conducted two Pearson correlation analyses. SF was negatively correlated with *A_a_* and *A_f_* at posttest 1 (*r* = −0.884, *p* = 0.047; *r* = −0.889, *p* = 0.044, respectively); similarly, SF had a significant negative correlation with *A_a_* and *A_f_* at posttest 2 (*r* = −0.879, *p* = 0.049; *r* = −0.93, *p* = 0.022, respectively). These results indicated that the changes in internal additive noise and perceptual template were larger as SF increased.

### The relationship among improvements in SE, VA, and CS after SMILE

To determine the relation of visual indicators, we calculated improvements in SE, VA, and CS at posttest 1 (values at posttest 1 minus those at pretest) and posttest 2 (values at posttest 2 minus those at pretest). CS was represented by AULCSF in the zero-noise condition. VA was measured by the LogMAR vision chart, and a lower LogMAR denoted better VA. At posttest 1, the correlation between SE improvement and VA improvement was strong (*r* = −0.569, *p* = 0.034). AULCSF improvement was not related to SE improvement (*r* = 0.415, *p* = 0.14). At posttest 2, increased SE was not correlated with increased VA but was positively correlated with AULCSF improvement (*r* = −0.505, *p* = 0.065; *r* = 0.632, *p* = 0.015, respectively). In summary, at posttest 1, the larger the SE improvement was, the larger the observed VA improvement was; at posttest 2, a larger SE improvement predicted a larger AULCSF increase.

Furthermore, linear regression models based on correlative analysis were constructed. At posttest 1, VA improvement (Y) and SE improvement (X) were significantly correlated, which fitted the regression equation Y = −0.291–0.122X. Similarly, at posttest 2, according to the significant correlation between increased AULCSF (Y) and increased SE (X), the regression equation was established as Y = 1.583 + 0.477X.

## Discussion

This study demonstrated that SMILE led to improvements in vision, including changes in variables (i.e., VA, SE, and CS) and the relationship among them; then, this study investigated the mechanisms of perception alteration with the PTM framework. CS improvements after SMILE were observed and dependent on external noise levels and SF conditions. CS recovery required time. Furthermore, SMILE reduced internal additive noise and enhanced the perceptual template, and both changes were SF dependent.

Prior studies focused on the influence of SMILE and found improvements in VA and SE; however, the trend of CS change was uncertain. To clarify the CS change, the current study applied a Bayesian Algorithm based procedure called qCSF. Compared with traditional tests that need to measure CS at a limited SF and require a long test time, qCSF extends the adaptive stimulus search to both grating stimulus dimensions (frequency and contrast), which makes it less time-consuming (Lesmes, 2010) and avoids the impact of fatigue. It also had advantages of high precision and accuracy. Based the qCSF, a clear pattern of increased CS at the 1st and 7th postoperative days versus the SF function was obtained. Generally speaking, SMILE improved CS at most SFs. In addition, CS increased with time; specifically, CS on the 7th day 7 days after SMILE was better than that on the 1st day after the surgery.

The inclusion of external noise when analyzing CSF is another innovation of this research. The AULCSF is an overall assessment of CS at 10 SFs. We found that AULCSF increased with time (i.e., from preoperative to postoperative) under different external noise levels, showing the effectiveness of SMILE and the quick recovery of wounds. In addition, AULCSF improvements decreased as external noise increased. Interestingly, when external noise was present, the CS at low SF (e.g., 0.5 cpd) became significantly worse on the 1st day after SMILE and then was restored to normal levels on the 7th day after SMILE. This suggested that healing wounds requires time, and this phenomenon was sensitive to external noise levels.

In the current study, we analyzed the underlying mechanisms of visual perception changes. The perceptual system is limited by an equivalent internal noise source; in other words, internal additive noise is associated with the efficiency of the visual processor. We found that CS improvements after SMILE could be attributed to lower internal additive noise and an enhanced perceptual template. In addition, the changes in internal additive noise and perceptual template were larger as SF increased. However, the PTM could not be used to analyze the data at intermediate and high SFs (e.g., >2 cpd). This is because the CS at those SFs of myopia subjects was quite poor before SMILE, and a floor effect was observed. In other words, CS improvement was underestimated when SFs were higher than 2 cpd.

Finally, although this study had the limitation of the relatively short follow-up time, our design mainly focused on recovery in the early stage after SMILE. According to previous studies, refractive stability was observed by one week postoperatively, which meant that vision tended to be stable instead of changing with time (Sekundo et al., 2011; Shah et al., 2011). Thus, we measured CS, VA, and SE at three time points (before SMILE, 1 day after the surgery and 7 days after the surgery) and found that SE was comparable in two postoperative measurements, which was consistent with the existing results. Similarly, VA had no significant difference between two postoperative measurements; however, CS increased significantly across three time points, indicating that CS could be a sensitive indicator to use to measure changes in visual function relative to VA, especially in a low-brightness environment. CS measurements reflect the whole contrast and SF, unlike VA, which is acquired under high contrast; for example, CS loss impaired driving performance, while VA loss had a weaker relation with poor driving behavior, such as low response to road hazards (Wood and Owens, 2005). In addition, we found that SE improvement could effectively predict increased VA on the 1st postoperative day; however, on the 7th postoperative day, increased SE positively predicted increased AULCSF on the 7th postoperative day. Hence, both VA and CS should be taken into consideration when estimating the effect of myopia surgery.

In summary, SMILE is a promising newer technique that results in effective improvements in vision characteristics, including SE, VA, and CS; thus, the above factors should be taken into account when assessing visual changes. In addition to the improvements in vision, the influence of SMILE on quality of life is an important issue that should be explored in future studies.

## Data availability statement

The raw data supporting the conclusions of this article will be made available by the authors, without undue reservation.

## Ethics statement

The studies involving human participants were reviewed and approved by the Ethics Committee of Hebei Normal University and the Ethics Committee of Shijiazhuang People’s Hospital. The patients/participants provided their written informed consent to participate in this study.

## Author contributions

PZ and HZ design and collected the data. PY and PZ analyzed the data. PY, PZ, and HZ wrote the manuscript. All authors revised the manuscript, contributed to the article, and approved the submitted version.

## Funding

This work was supported by the Social Science Foundation of Hebei Province (HB20JY020, HB14JY031 and HB10VJY032 to ZW), Science and Technology Project of Hebei Province (22556202K to ZW), Social Science Development Project of Hebei Province (20220202304 to ZW) and Natural Science Foundation of Hebei Province (C2012205046 to ZW and C2021205005 to PZ).

## Conflict of interest

The authors declare that the research was conducted in the absence of any commercial or financial relationships that could be construed as a potential conflict of interest.

## Publisher’s note

All claims expressed in this article are solely those of the authors and do not necessarily represent those of their affiliated organizations, or those of the publisher, the editors and the reviewers. Any product that may be evaluated in this article, or claim that may be made by its manufacturer, is not guaranteed or endorsed by the publisher.

## Supplementary material

The Supplementary material for this article can be found online at: https://www.frontiersin.org/articles/10.3389/fnins.2023.1132681/full#supplementary-material
