# Supplementary material for: The effect of small incision lenticule extraction on contrast sensitivity
Source: Front Neurosci. 2023 Apr 12;17:1132681. doi: 10.3389/fnins.2023.1132681 (PMC10130440; doi:10.3389/fnins.2023.1132681)
Supplement: Supplementary file 1 [file Data_Sheet_1.docx]

***Supplementary Material***

**The effect of small incision lenticule extraction on contrast sensitivity**

**Pinqing Yue^1^, Zeng Wang^2^, Di Wu^3^, Hua Zhang^4*^, Pan Zhang^1*^**

^1^ Department of Psychology, Hebei Normal University, Shijiazhuang, China, 050024;

^2^ Department of Psychology, Hebei Medical University, Shijiazhuang, China, 050017;

^3^ Department of Medical Psychology, Air Force Medical University, Xi’an, China, 710032;

^4^ Department of Ophthalmology, Shijiazhuang People’s Hospital, Shijiazhuang, China, 050025;

**^*^Corresponding:**

Pan Zhang, [zhang2005pan@126.com](mailto:zhang2005pan@126.com);

Hua Zhang, [zhanghua_dr@126.com](mailto:zhanghua_dr@126.com)

# 1 Supplementary Tables

| **Table S1.** P-values for Comparison of CS between different time points at each SF | | | | |
| --- | --- | --- | --- | --- |
| external noise | SF (cpd) | time point (I) | time point (J) | *AD*(I-J) |
| zero | 0.5 | posttest 1 | pretest | 0.315^**^ |
|  |  | posttest 2 | pretest | 0.504^***^ |
|  |  |  | posttest 1 | 0.189^*^ |
|  | 0.67 | posttest 1 | pretest | 0.423^***^ |
|  |  | posttest 2 | pretest | 0.646^***^ |
|  |  |  | posttest 1 | 0.224^**^ |
|  | 1 | posttest 1 | pretest | 0.644^***^ |
|  |  | posttest 2 | pretest | 0.921^***^ |
|  |  |  | posttest 1 | 0.276^***^ |
|  | 1.33 | posttest 1 | pretest | 0.756^***^ |
|  |  | posttest 2 | pretest | 1.072^***^ |
|  |  |  | posttest 1 | 0.317^***^ |
|  | 2 | posttest 1 | pretest | 0.833^***^ |
|  |  | posttest 2 | pretest | 1.210^***^ |
|  |  |  | posttest 1 | 0.377^***^ |
|  | 2.67 | posttest 1 | pretest | 0.711^***^ |
|  |  | posttest 2 | pretest | 1.131^***^ |
|  |  |  | posttest 1 | 0.420^***^ |
|  | 4 | posttest 1 | pretest | 0.411^***^ |
|  |  | posttest 2 | pretest | 0.807^***^ |
|  |  |  | posttest 1 | 0.397^***^ |
|  | 5.33 | posttest 1 | pretest | 0.206^**^ |
|  |  | posttest 2 | pretest | 0.564^***^ |
|  |  |  | posttest 1 | 0.358^***^ |
|  | 8 | posttest 1 | pretest | 0.052 |
|  |  | posttest 2 | pretest | 0.282^**^ |
|  |  |  | posttest 1 | 0.229^**^ |
|  | 16 | posttest 1 | pretest | 0.000 |
|  |  | posttest 2 | pretest | 0.005 |
|  |  |  | posttest 1 | 0.005 |
| low | 0.5 | posttest 1 | pretest | -0.190^*^ |
|  |  | posttest 2 | pretest | -0.114 |
|  |  |  | posttest 1 | 0.075 |
|  | 0.67 | posttest 1 | pretest | -0.121 |
|  |  | posttest 2 | pretest | -0.030 |
|  |  |  | posttest 1 | 0.091 |
|  | 1 | posttest 1 | pretest | 0.139 |
|  |  | posttest 2 | pretest | 0.290^**^ |
|  |  |  | posttest 1 | 0.151^*^ |
|  | 1.33 | posttest 1 | pretest | 0.343^**^ |
|  |  | posttest 2 | pretest | 0.511^***^ |
|  |  |  | posttest 1 | 0.168^*^ |
|  | 2 | posttest 1 | pretest | 0.571^***^ |
|  |  | posttest 2 | pretest | 0.757^***^ |
|  |  |  | posttest 1 | 0.186^**^ |
|  | 2.67 | posttest 1 | pretest | 0.616^***^ |
|  |  | posttest 2 | pretest | 0.816^***^ |
|  |  |  | posttest 1 | 0.200^**^ |
|  | 4 | posttest 1 | pretest | 0.484^***^ |
|  |  | posttest 2 | pretest | 0.718^***^ |
|  |  |  | posttest 1 | 0.234^**^ |
|  | 5.33 | posttest 1 | pretest | 0.338^***^ |
|  |  | posttest 2 | pretest | 0.594^***^ |
|  |  |  | posttest 1 | 0.256^**^ |
|  | 8 | posttest 1 | pretest | 0.165^**^ |
|  |  | posttest 2 | pretest | 0.368^***^ |
|  |  |  | posttest 1 | 0.203^*^ |
|  | 16 | posttest 1 | pretest | 0.001 |
|  |  | posttest 2 | pretest | 0.057 |
|  |  |  | posttest 1 | 0.057 |
| high | 0.5 | posttest 1 | pretest | -0.186^**^ |
|  |  | posttest 2 | pretest | -0.080 |
|  |  |  | posttest 1 | 0.106^**^ |
|  | 0.67 | posttest 1 | pretest | -0.172^***^ |
|  |  | posttest 2 | pretest | -0.069 |
|  |  |  | posttest 1 | 0.102^**^ |
|  | 1 | posttest 1 | pretest | -0.043 |
|  |  | posttest 2 | pretest | 0.042 |
|  |  |  | posttest 1 | 0.086^*^ |
|  | 1.33 | posttest 1 | pretest | 0.122^*^ |
|  |  | posttest 2 | pretest | 0.210^**^ |
|  |  |  | posttest 1 | 0.088^**^ |
|  | 2 | posttest 1 | pretest | 0.300^***^ |
|  |  | posttest 2 | pretest | 0.413^***^ |
|  |  |  | posttest 1 | 0.114^***^ |
|  | 2.67 | posttest 1 | pretest | 0.368^***^ |
|  |  | posttest 2 | pretest | 0.502^***^ |
|  |  |  | posttest 1 | 0.134^***^ |
|  | 4 | posttest 1 | pretest | 0.352^***^ |
|  |  |  | posttest 2 | -0.167 |
|  |  | posttest 2 | pretest | 0.519^***^ |
|  |  |  | posttest 1 | 0.167^***^ |
|  | 5.33 | posttest 1 | pretest | 0.285^***^ |
|  |  | posttest 2 | pretest | 0.467^***^ |
|  |  |  | posttest 1 | 0.182^**^ |
|  | 8 | posttest 1 | pretest | 0.178^**^ |
|  |  | posttest 2 | pretest | 0.335^***^ |
|  |  |  | posttest 1 | 0.158^*^ |
|  | 16 | posttest 1 | pretest | 0.037 |
|  |  | posttest 2 | pretest | 0.096^*^ |
|  |  |  | posttest 1 | 0.060 |

*Note. AD* represents average difference. ^*^*p* < 0.05; ^**^*p* < 0.01; ^***^*p* < 0.001.

| **Table S2.** P-values for comparison of CS improvement between different SFs | | | | | | | | | | | |
| --- | --- | --- | --- | --- | --- | --- | --- | --- | --- | --- | --- |
| time point | external noise | SF (cpd) | 0.67 | 1 | 1.33 | 2 | 2.67 | 4 | 5.33 | 8 | 16 |
| posttest 1 | zero | 0.5 | 0.002 | 0.000 | 0.000 | 0.001 | 0.013 | 0.463 | 0.360 | 0.010 | 0.001 |
|  |  | 0.67 |  | 0.000 | 0.000 | 0.004 | 0.056 | 0.928 | 0.096 | 0.002 | 0.000 |
|  |  | 1 |  |  | 0.014 | 0.112 | 0.637 | 0.102 | 0.006 | 0.000 | 0.000 |
|  |  | 1.33 |  |  |  | 0.340 | 0.680 | 0.006 | 0.000 | 0.000 | 0.000 |
|  |  | 2 |  |  |  |  | 0.006 | 0.000 | 0.000 | 0.000 | 0.000 |
|  |  | 2.67 |  |  |  |  |  | 0.000 | 0.000 | 0.000 | 0.000 |
|  |  | 4 |  |  |  |  |  |  | 0.000 | 0.000 | 0.000 |
|  |  | 5.33 |  |  |  |  |  |  |  | 0.003 | 0.009 |
|  |  | 8 |  |  |  |  |  |  |  |  | 0.131 |
|  |  |  |  |  |  |  |  |  |  |  |  |
|  | low | 0.5 | 0.093 | 0.005 | 0.000 | 0.000 | 0.000 | 0.000 | 0.000 | 0.000 | 0.039 |
|  |  | 0.67 |  | 0.001 | 0.000 | 0.000 | 0.000 | 0.000 | 0.000 | 0.001 | 0.081 |
|  |  | 1 |  |  | 0.000 | 0.000 | 0.000 | 0.001 | 0.071 | 0.813 | 0.137 |
|  |  | 1.33 |  |  |  | 0.000 | 0.001 | 0.114 | 0.962 | 0.141 | 0.003 |
|  |  | 2 |  |  |  |  | 0.180 | 0.168 | 0.018 | 0.001 | 0.000 |
|  |  | 2.67 |  |  |  |  |  | 0.006 | 0.002 | 0.000 | 0.000 |
|  |  | 4 |  |  |  |  |  |  | 0.000 | 0.000 | 0.000 |
|  |  | 5.33 |  |  |  |  |  |  |  | 0.000 | 0.000 |
|  |  | 8 |  |  |  |  |  |  |  |  | 0.006 |
|  |  |  |  |  |  |  |  |  |  |  |  |
|  | high | 0.5 | 0.382 | 0.010 | 0.001 | 0.000 | 0.000 | 0.000 | 0.000 | 0.000 | 0.000 |
|  |  | 0.67 |  | 0.002 | 0.000 | 0.000 | 0.000 | 0.000 | 0.000 | 0.000 | 0.000 |
|  |  | 1 |  |  | 0.000 | 0.000 | 0.000 | 0.000 | 0.000 | 0.001 | 0.060 |
|  |  | 1.33 |  |  |  | 0.000 | 0.000 | 0.001 | 0.021 | 0.410 | 0.142 |
|  |  | 2 |  |  |  |  | 0.003 | 0.228 | 0.785 | 0.057 | 0.000 |
|  |  | 2.67 |  |  |  |  |  | 0.583 | 0.073 | 0.002 | 0.000 |
|  |  | 4 |  |  |  |  |  |  | 0.003 | 0.000 | 0.000 |
|  |  | 5.33 |  |  |  |  |  |  |  | 0.000 | 0.000 |
|  |  | 8 |  |  |  |  |  |  |  |  | 0.002 |
|  |  |  |  |  |  |  |  |  |  |  |  |
| posttest 2 | zero | 0.5 | 0.000 | 0.000 | 0.000 | 0.000 | 0.000 | 0.075 | 0.714 | 0.131 | 0.000 |
|  |  | 0.67 |  | 0.000 | 0.000 | 0.000 | 0.003 | 0.322 | 0.619 | 0.023 | 0.000 |
|  |  | 1 |  |  | 0.002 | 0.018 | 0.143 | 0.470 | 0.041 | 0.001 | 0.000 |
|  |  | 1.33 |  |  |  | 0.093 | 0.594 | 0.049 | 0.001 | 0.000 | 0.000 |
|  |  | 2 |  |  |  |  | 0.066 | 0.000 | 0.000 | 0.000 | 0.000 |
|  |  | 2.67 |  |  |  |  |  | 0.000 | 0.000 | 0.000 | 0.000 |
|  |  | 4 |  |  |  |  |  |  | 0.000 | 0.000 | 0.000 |
|  |  | 5.33 |  |  |  |  |  |  |  | 0.000 | 0.000 |
|  |  | 8 |  |  |  |  |  |  |  |  | 0.002 |
|  |  |  |  |  |  |  |  |  |  |  |  |
|  | low | 0.5 | 0.027 | 0.000 | 0.000 | 0.000 | 0.000 | 0.000 | 0.000 | 0.001 | 0.121 |
|  |  | 0.67 |  | 0.000 | 0.000 | 0.000 | 0.000 | 0.000 | 0.000 | 0.003 | 0.351 |
|  |  | 1 |  |  | 0.000 | 0.000 | 0.000 | 0.000 | 0.011 | 0.485 | 0.032 |
|  |  | 1.33 |  |  |  | 0.000 | 0.001 | 0.015 | 0.360 | 0.167 | 0.000 |
|  |  | 2 |  |  |  |  | 0.048 | 0.334 | 0.016 | 0.000 | 0.000 |
|  |  | 2.67 |  |  |  |  |  | 0.005 | 0.001 | 0.000 | 0.000 |
|  |  | 4 |  |  |  |  |  |  | 0.000 | 0.000 | 0.000 |
|  |  | 5.33 |  |  |  |  |  |  |  | 0.000 | 0.000 |
|  |  | 8 |  |  |  |  |  |  |  |  | 0.000 |
|  |  |  |  |  |  |  |  |  |  |  |  |
|  | high | 0.5 | 0.526 | 0.018 | 0.001 | 0.000 | 0.000 | 0.000 | 0.000 | 0.000 | 0.021 |
|  |  | 0.67 |  | 0.002 | 0.000 | 0.000 | 0.000 | 0.000 | 0.000 | 0.000 | 0.012 |
|  |  | 1 |  |  | 0.000 | 0.000 | 0.000 | 0.000 | 0.000 | 0.000 | 0.289 |
|  |  | 1.33 |  |  |  | 0.000 | 0.000 | 0.000 | 0.000 | 0.056 | 0.057 |
|  |  | 2 |  |  |  |  | 0.001 | 0.016 | 0.247 | 0.183 | 0.000 |
|  |  | 2.67 |  |  |  |  |  | 0.530 | 0.338 | 0.005 | 0.000 |
|  |  | 4 |  |  |  |  |  |  | 0.005 | 0.000 | 0.000 |
|  |  | 5.33 |  |  |  |  |  |  |  | 0.000 | 0.000 |
|  |  | 8 |  |  |  |  |  |  |  |  | 0.000 |
